# Orbital endoscopic decompression in Graves Ophthalmopathy

**DOI:** 10.1016/S1808-8694(15)30069-0

**Published:** 2015-10-19

**Authors:** Wilma Terezinha Anselmo Lima, Mateus Perches, Fabiana Cardoso Pereira Valera, Ricardo Cassiano Demarco

**Affiliations:** a Associate Professor.; b MD, Resident of Otorhinolaryngology - Department of Otorhinolaryngology, Ophthalmology and Head and Neck Surgery - FMRP-USP.; c Assistant physician - University Hospital - Department of Otorhinolaryngology, Ophthalmology and Head and Neck Surgery - FMRP-USP.; d Assistant physician - University Hospital - Department of Otorhinolaryngology, Ophthalmology and Head and Neck Surgery - FMRP-USP.

**Keywords:** Graves’ disease, endoscopic orbital decompression, proptosis

## Abstract

Graves’ disease may lead to exophthalmos that is cosmetically unacceptable or causes visual loss. This has been managed surgically by external orbital decompression. However, a new minimally invasive endoscopic orbital decompression technique is now possible, with resection of the medial and posterior portion of the orbital limits requiring no cutaneous or gingival incisions. This technique produces decompression comparable to that obtained by external techniques. Endoscopic orbital decompression is a safe and effective procedure for the treatment of thyroid orbitopathy.

## INTRODUCTION

Graves’ Ophthalmopathy is an autoimmune disease characterized by deposition of antithyroglobulin immune complexes on the extra-ocular muscles. These complexes induce an inflammatory response through lymphocytes, mast cells and plasmatic cells, causing edema and fibrosis later on, with hypertrophy of the extra-ocular muscles - especially the inferior and middle rectus muscles and orbitary fat. Moreover, there is a stimulation of fibroblast-myogenic activity, resulting in an increase in orbital volume, causing proptosis, mainly. It is more common to women than men, with average age between 20 and 40 years^1^, ^2^, ^3^.

The most common clinical signs are proptosis - in different levels of intensity, asymmetrical or bilateral; and diplopia, because of the abnormal and asymmetrical mobility of extra-ocular muscles infiltrated by inflammatory cells. In more severe cases there is keratoconjunctivities, eyelid retraction and corneal ulcerations because of insufficient eyelid closure, and finally, optical neuropathy caused by a compression on the posterior third of ophthalmic nerve, at the orbital apex.

Orbital involvement occurs in about 50% of the patients with Graves’ disease, but only 5 to 10% of these develop severe ophthalmopathy.

The disease evolves slowly but progressively until it stabilizes, there are, however, rare cases of spontaneous resolution. Notwithstanding, those patients who develop severe proptosis or optical neuropathy require clinical or surgical treatment.

About two-thirds of the patients respond to other treatment modalities, specially if treated in the acute phase, treated with steroids, orbital radiation (which is not efficient for proptosis and diplopia), immunesupressors and plasmapheresis. The acute phase lasts from 6 to 18 months, with orbital inflammation and congestion, increase in intraorbitary volume causing proptosis with anterior shifting of the eye ball. It evolves to a chronic and stable phase which may start from 18 months to 3 years after orbitopathy onset. In this phase, the fibrotic process sets in.

The orbitopathy seems to follow a different pathway from the thyroid treatment, and normally does not bear relation with the thyroid abnormalities. It may precede, follow or even occur after hyperthyroidism, or even without clear hyperthyroidism.

Surgical treatment for Graves’ ophthalmopathy is used to treat the consequences of the disease and, therefore, is done during the disease stable phase. It is indicated whenever there are symptoms of ocular surface exposure, optical neuropathy or for cosmetic reasons. It includes repairing strabismus, eyelid retraction and orbital decompression to correct exophthalmia6. The decompression major goals are to open more room to accommodate orbital content, thus reducing orbital tissue pressure; restore vision, allow the functioning of the extra-ocular muscles, eyelid closure and, consequently, reduce proptosis. There are cosmetic improvements. Different approaches have been described for orbital decompression: Lateral orbitotomy, frontal craniotomy, external fronto-ethmoidectomy, orbital transnasal decompression^7^, ^8^, ^9^. Through these approached we may remove the orbital medial or lateral walls, orbital floor, both the floor and medial wall, both floor and lateral wall, and anterior cranial fossa. Walsh-Ogura^7^ procedure, which uses a transnasal approach was the one most employed. Through a broad maxillary antrostomy we are able to remove the orbital floor, sparing the infra-orbitary nerve, followed by a transnasal ethmoidectomy and decompression of the medial orbital wall. However, it is an approach associated to Caldwell-Luc’s procedure morbidity and may result in inferior shifting of the eye ball, with access limitations for the decompression of the orbital apex.

With the development of endoscopic surgery, the transnasal removal of the lamina papyracea has caused proptosis reduction comparable to the results of other approaches, with advantages. Described by Kennedy et al.^10^ and Michel et al.^4^. It allows excellent view without the need for external incisions, with less morbidity and a more efficient approach for the optical neuropathy. Our goal with this paper is to describe the surgical technique, focusing on its advantages and disadvantages.

### Preoperative:

Basically, besides routine evaluation carried out for any type of surgery, endoscopic assessment and radiology are paramount. Through endoscopy we may see alterations such as septal deviations and the presence of infections, which may be treated clinically or during the surgical procedure. The coronal slice in the CT scan is useful to access how aerated the ethmoid is, the integrity and slope of the ethmoid ceiling, as well as the orbital medial wall (it is important to evaluate the height of the posterior ethmoid sinus and its relation with the maxillary ceiling and determine whether the posterior ethmoid expands posteriorly and superiorly, involving the optical nerve and or the internal carotid artery), the thickness of the orbital floor (the thicker it is, the harder it is to remove it endoscopically through middle meatal antrostomy), the skull base situation. The axial view is useful to show the relation between the posterior ethmoid artery, the optical nerve and the orbital apex. Both views show the hypertrophy of the extrinsic eye muscles (Figure 1a, Figure 1b).

**Figure 1a:**
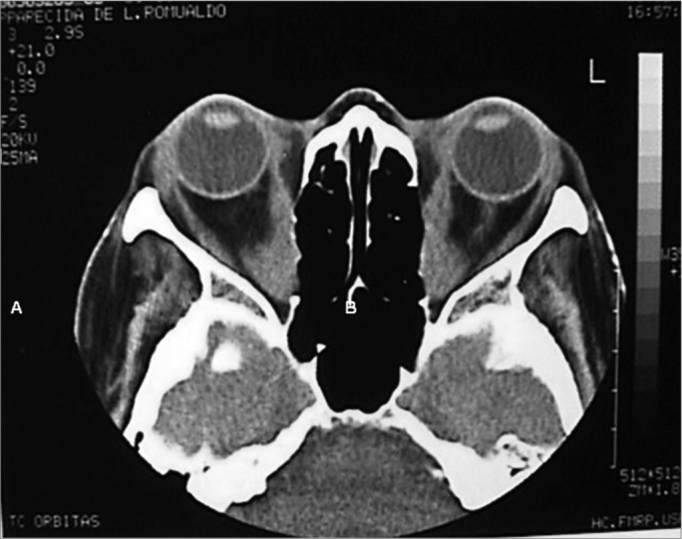
CT scans, axial views (A) of a patient with Graves’ ophthalmopathy, showing the hypertrophy of the extrinsic ocular muscles.

**Figure 1b:**
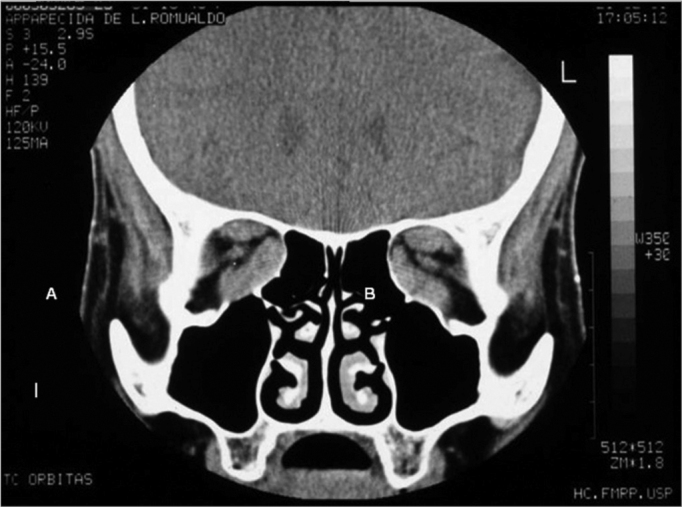
CT scans, coronal views (B) of a patient with Graves’ ophthalmopathy, showing the hypertrophy of the extrinsic ocular muscles.

## SURGICAL TECHNIQUE

**Figure 2:**
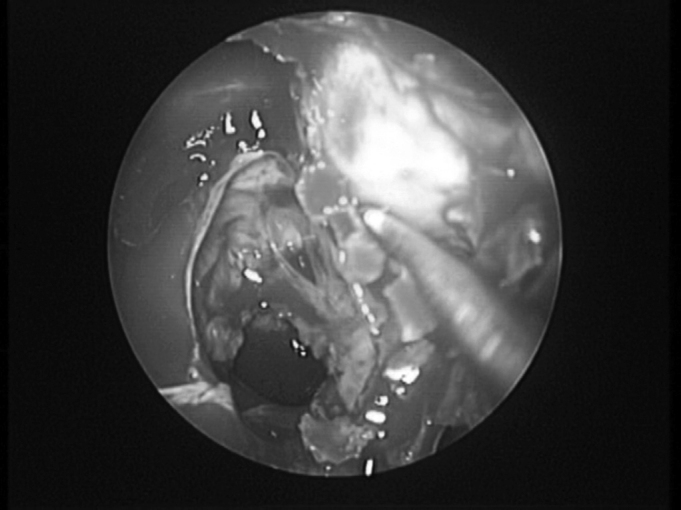
Removal of the lamina papyracea in a left side orbital decompression.

Under general anesthesia, the patient is initially positioned in dorsal decubitus, with the head slightly tilted upwards, in 30º. We inject the anesthetic agent on the lateral wall placing cotton balls with vasoconstrictor agents in the nasal cavities. If there is septum deviation we correct it prior to the main endoscopic procedure. The orbital decompression (OD) starts in the unciform process incision, with complete ethmoidectomy^11^. We proceed with middle meatotomy as broadly as possible, in order to visualize the orbital floor, down to the posterior maxillary wall, enough to accommodate the orbital content and prevent obstructive maxillary sinusitis. We use the 45º endoscope to see the infraorbitary nerve in its canal, along the ceiling of the maxillary sinus, because this nerve represents the lateral limit for bone removal. Following that, we proceed with a transethmoidal sphenoidectomy, and anteriorly, we open the frontal recess. One should pay special attention to Onodi’s cells, where the optical nerve may bulge. The anterior limit corresponds to the frontal maxillary process, close to the nasolacrimal duct. The superior limit corresponds to the floor of the anterior cranial fossa, where the ethmoidal arteries represent an important anatomical reference point. Inferiorly, the insertion of the inferior turbinate is the limit. The middle turbinate may be removed in order to optimize the orbital content prolapse. The lamina papyracea is then skeletonized and removed with the periosteum elevator, which easily separates it from the periorbital tissue (Figure 2).

The lamina is carefully removed in order to avoid lacerations in the periorbital area, where there may be fat tissue herniations which may impair the periorbital skeletonization. We proceed with the dissection all the way to the posterior ethmoid bundle, close to the optical nerve, where the bone is thicker. Here we find the Zinn annulus, where the extra-ocular muscles are inserted, and certainly the optical nerve path. With the 45º endoscope we remove the orbital floor down to the infra-orbital vascular-nervous bundle, which is our lateral dissection limit. We dissect as posteriorly as possible.

**Figure 3:**
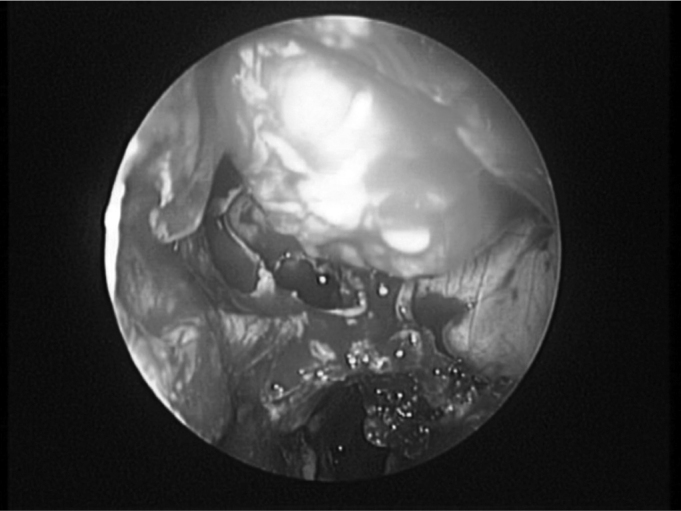
Extrusion of the orbital content after left side orbit endoscopic decompression.

Once the periorbit is fully dissected and exposed, we make an incision in order to allow a free fat prolapse from the periorbital tissue. We proceed making two to four longitudinal, postero-anterior and infero-superior periorbital incisions. This sequence reduces the chance of the herniated fat impair the surgeon’s view. One should be careful with the superficial unciform process removal in order to avoid penetrating the intraorbitary content. At the end of the procedure there is fat and orbital content extrusion (Figure 3). If necessary, we may proceed with lateral decompression at this stage, because through the medial decompression, it will be easy to expose the lateral bone wall. There is no need for nasal packing. The patient may be discharged on the next day following the procedure, with oral antibiotics (amoxicillin for 10 days) and saline solution nasal spray.

## DISCUSSION

The progress of endoscopic procedures has opened new possibilities for otorhinolaryngologists to work in different areas that were considered difficult, such as dacriocistorrinostomy, orbital decompression and optical nerve decompression, in a safe and minimally invasive way.

This technique was first described by Kennedy et al. in the early 90’s^10^. The endoscopic approach allows an excellent view for a safe removal of the medial and inferior orbital wall, specially in the regions of the ethmoidal ceiling and orbital apex. The endoscopic orbital decompression is based on a broad middle meatotomy in order to allow broad view of the orbital floor.

As to the transantral approach, the endoscopic orbital decompression does not cause hypoesthesia secondary to infra-orbitary nerve lesion, and causes fewer incidences of hypoglobus, thus allowing for a shorter hospital stay when compared to the traditional Walsh-Ogura^7^ procedure. It also allows a more effective decompression of the orbit apex, specially indicated in cases of orbital neuropathy.

The most difficult surgical step is the removal of the medial portion of the orbital floor, medially to the infraorbitary nerve, due to an excessive thickness that may be present on the floor; however it may be previously evaluated through the CT scan. Bone resections laterally to the infraorbitary cause little proptosis reduction and increase the incidence of hypoglobus and diplopia. Michel et al.^4^ also described the bilateral removal of the middle conchae during decompression, aiming at increasing the area for orbit content extrusion.

Endoscopically we have been able to achieve variable proptosis retraction, between 3.2 to 4.7 millimeters, as we can see on table^14^, ^10^, ^12^, ^13^. However, greater retractions may be reached if external approaches are associated, such as the removal of the lateral orbital wall.

Among the disadvantages, we mention a higher incidence of diplopia, which may be surgically corrected later on, and the potential development of sinusitis or secondary mucocele^14^, ^15^, ^16^, ^17^, ^18^.

The most frequently reported complications with this procedure are sinusitis or frontal or maxillary mucocele, CSF fistulas, lesion of the nasolacrimal duct, strabismus and diplopia, which may occur in 15 to 60% of the cases. The latter may have spontaneous resolution in three to four weeks, but it may also appear later on, due to disease progress. In these cases, there is the need for strabismus correction.

**Table 1:** Reported series of proptosis retraction through endoscopiconly orbital decompression.

Series	# of orbits	Average proptosis reduction
Kennedy et al.^10^	5	4,7mm
Michel et al.^4^	12	3,3mm
Lund et al. 12	24	4,4mm
Metson & Shore^13^	6	3,2mm

## FINAL COMMENTS

In the past this procedure was carried out externally, but currently the orbital decompression may be performed through an endoscopic approach, a minimally invasive procedure, allowing the removal of the infero-medial all without external cuts. It is a safe procedure for the treatment of thyroid orbitopathy, bearing less morbidity, avoiding lesions to the nasolacrimal and nasofrontal duct, or the infraorbitary nerve, allowing a proptosis reduction of 3 to 4 mm. This technique allows a maximum decompression of the orbital apex in cases or orbital neuropathy. However, it is necessary to have a trained surgeon in order to achieve good results and avoid complications.

## References

[1] Weetman AP (1991). Thyroid-associated eye disease: Pathophysiology. Lancet.

[2] Mc Cord Jr CD. (1985). Current trends in orbital decompression. Ophthalmology.

[3] Naffizer HC (1931). Progressive exophathalmos following thyroidectomy: Its pathology and treatment. Ann Surg.

[4] Michel O (1991). Endoskopish kontrolierte endonasale orbitadekompression beim malignen ophtalmus. Laryngorhinootologie.

[7] Walsh TE, Ogura JH (1957). Transantral orbital decompression for malignant exophtalmos. Laryngoscope.

[8] Hirsch O (1950). Surgical decompression of exophthalmos. Arch Otolaryngol Head Neck Surg.

[9] Sewall EC (1936). Operative control of progressive exophthalmos. Arch Otolaryngol Head Neck Surg.

[10] Kennedy DW, Goodstein MZ, Miller NR, Zinreich SJ (1990). Endoscopic transnasal orbital decompression. Arch Otolaryngol Head Neck Surg.

[11] Kennedy DW (1985). Functional endoscopic sinus surgery technique. Arch Otolaryngol Head Neck Surg.

[12] Lund VJ, Adams G (1997). Orbital decompression for thyroid eye disease: a comparison of external and endoscopic techniques. J Laryngol Otol.

[13] Metson R, Shore JW (1994). Endoscopic orbital decompression. Laryngoscope.

[14] Henrich DH, Kennedy DW, Stankiewcz JA (1995). Advanced Endocopic Sinnus Surgery.

[15] Sacks EH, Anand VK, Lisman RD, Anand VK, Panje ER (1993). Practical Endoscopic Sinnus Surgery.

[16] Hwang PH, Kennedy DW, Stamm Ac, Draf W. (2000). Micro-Endoscopic Surgery of the Paranasal Sinuses And the Skull Base Springer Berlin.

[17] Luxenberger W, Stammberger H, Jebeles JA, Walch C (1998). Endoscopic optic nerve decompression: The Graz experience. The Laryngoscope.

[18] De Santo LW (1980). The total rehabilitation of Graves ophthalmopathy. Laryngoscope.

